# Leveraging transfer learning for predicting total knee arthroplasty failure from post‐operative radiographs

**DOI:** 10.1002/jeo2.70097

**Published:** 2024-12-11

**Authors:** Anna Corti, Sarah Galante, Rebecca Rauch, Katia Chiappetta, Valentina Corino, Mattia Loppini

**Affiliations:** ^1^ Department of Electronics, Information and Bioengineering Politecnico di Milano Milan Milan Italy; ^2^ Humanitas University, Pieve Emanuele MI Italy; ^3^ IRCCS Humanitas Research Hospital Rozzano Milan Italy; ^4^ Cardio Tech‐Lab Centro Cardiologico Monzino IRCCS Milan Milan Italy; ^5^ Department of Biomedical Sciences, Humanitas University Pieve Emanuele Milan Italy

**Keywords:** deep learning, image classification, prosthesis revision, total knee arthroplasty, transfer learning

## Abstract

**Purpose:**

The incidence of both primary and revision total knee arthroplasty (TKA) is expected to rise, making early recognition of TKA failure crucial to prevent extensive revision surgeries. This study aims to develop a deep learning (DL) model to predict TKA failure using radiographic images.

**Methods:**

Two patient cohorts who underwent primary TKA were retrospectively collected: one was used for the model development and the other for the external validation. Each cohort encompassed failed and non‐failed subjects, according to the need for TKA revision surgery. Moreover, for each patient, one anteroposterior and one lateral radiographic view obtained during routine TKA follow‐up, were considered. A transfer learning fine‐tuning approach was employed. After pre‐processing, the images were analyzed using a convolutional neuronal network (CNN) that was originally developed for predicting hip prosthesis failure and was based on the Densenet169 pre‐trained on Imagenet. The model was tested on 20% of the images of the first cohort and externally validated on the images of the second cohort. Metrics, such as accuracy, sensitivity, specificity and area under the receiving operating characteristic curve (AUC), were calculated for the final assessment.

**Results:**

The trained model correctly classified 108 out of 127 images in the test set, providing a classification accuracy of 0.85, sensitivity of 0.80, specificity of 0.89 and AUC of 0.86. Moreover, the model correctly classified 1547 out of 1937 in the external validation set, providing a balanced accuracy of 0.79, sensitivity of 0.80, specificity of 0.78 and AUC of 0.86.

**Conclusions:**

The present DL model predicts TKA failure with moderate accuracy, regardless of the cause of revision surgery. Additionally, the effectiveness of the transfer learning fine‐tuning approach, leveraging a previously developed DL model for hip prosthesis failure, has been successfully demonstrated.

**Level of Evidence:**

Level III, diagnostic study.

**Abbreviations**
AP: anteroposterior
AUC: area under the curve
CNN: convolutional neuronal network
CT: computed tomography
DL: deep learning
FN: false negative
FP: false positive
ML: machine learning
ROC: receiving operating characteristic curve
TKA: total knee arthroplasty
TN: true negative
TP: true positive

## INTRODUCTION

Total knee arthroplasty (TKA) is the gold‐standard orthopaedic surgery for treating patients with late‐stage knee osteoarthritis [4, 14]. The global number of TKA is rising due to the increased population longevity and the higher prevalence of knee arthritis [30].

A recent study in the United States reported that the annual volume of TKA increased by 156% between 2000 and 2019, and it is projected to rise by 139% by 2040 and 469% by 2060 [29]. The TKA procedure has a 10‐year cumulative revision rate ranging from 3.5% to 6%. The major causes of TKA revision include aseptic loosening, infection, instability, patellofemoral complication, and pain [10]. Consequently, along with the growth of global number of primary TKA procedures, an increase in revision TKA cases is also expected [28]. Recent projections estimate that revision TKA will increase by 149% by 2040 and by 520% by 2060 [28]. Revision TKA procedures are more complex, costly, and associated with decreased implant longevity and suboptimal patient‐reported outcomes when compared to primary TKA [2, 25]. The growing number of revisions presents significant challenges for the healthcare systems, making the early detection of primary TKA failure critically important in joint replacement surgery [2, 25, 28].

In orthopaedics, machine learning (ML) models have gained prominence, with earlier efforts focused on predicting various outcomes of TKA, including clinical results, opioid use, complications, length of hospital stay, costs, patient satisfaction, functional outcomes and the need for revision [6, 8, 11, 12, 13, 17, 32]. Artificial intelligence ML algorithms have also been investigated to predict the size of the final implant in TKA, while deep learning (DL) algorithms were applied to identify TKA candidates [15, 18]. Regarding TKA revision prediction, recent studies have developed ML and DL models based on clinical [1, 7] or radiographical data [16, 27]. For image‐based predictive models, both studies focused on predicting implant loosening: Shah et al. [27] utilized pre‐operative radiographs preceding primary TKA, achieving an accuracy of 85.8% on a test set of 138 patients, while Lau et al. [16] used post‐primary TKA radiographs, achieving an accuracy of 96.3% on a test set of 95 radiographs. However, to the best of our knowledge, no image‐based DL model has been proposed that considers all potential causes of TKA failure, rather than focusing only on implant loosening. Therefore, the primary aim of this study is to fill this gap by developing a model that accounts for all causes of TKA failure. Furthermore, given the challenge of requiring extensive data for DL models, we hypothesize that transfer learning will allow us to optimize existing models and adapt them to this new, related task with a limited dataset. Thus, given the success of the previously developed image‐based DL model, which achieved an accuracy of 0.97 in automatically identifying hip prosthesis failure from post‐operative conventional radiographs (following primary hip arthroplasty) [19, 23], this study also aims to investigate whether a transfer learning approach from the hip prosthesis failure model can accurately detect primary TKA failure from post‐operative conventional radiographs, obtained after primary TKA.

## METHODS

### Patient data set

For the purposes of this study, two cohorts of patients who underwent primary TKA surgery were retrospectively collected from the digital medical records at two high‐volume hospitals for prosthetic surgery between 2000 and 2021. One cohort was used for the DL model development, encompassing training and internal tests; the other was used for the external validation by evaluating the performance of the model on a new sample, different from that used for the development. The first cohort, used for the model developing and the internal testing, included 285 patients. Of these, 150 patients belonged to the failed group, with the definition of ‘failure’ determined by whether or not the patient required a TKA revision. The causes of failure included loosening, dislocation, periprosthetic fracture, polyethylene wear, instability and infection. Moreover, a control group of 135 patients (non‐failed patients) was randomly selected to obtain a balanced training dataset (at the image level). In the end, the first dataset included 298 and 304 images for the failed and non‐failed groups, respectively. Patients' selection was based on the availability of one anteroposterior (AP) and one lateral knee prosthesis radiograph during the follow‐up period for the non‐failed group and before TKA revision surgery for the failed group. If a patient of the non‐failed group underwent bilateral TKA surgery, both knees implants were utilized for the analysis.

The second cohort of patients, used for external validation, initially considered 1000 patients distinct from the first cohort, with a total of 2000 images. However, some patients were lost during analyses because radiographic images that were found twice or belonging to a follow‐up earlier than the sixth postoperative month were excluded. In the end, the second cohort consisted of 969 patients, subdivided into 165 failed TKA and 804 non‐failed, resulting in a total of 1937 images, divided into 329 failed and 1608 non‐failed.

Patients from the first cohort were split into training, validation and internal sets through a patient‐stratified approach: 20% (127 patients) were kept for the internal testing. The remaining 80% were further separated into 70%–30% for training (336 patients) and validation (139 patients), respectively. The number of image samples for internal testing, training and validation sets is reported in Table 1 for both failed and non‐failed groups, together with the external validation based on data from the second cohort.

**Table 1:** Patients' image data set representation and subdivision into testing, training, validation and external validation.

Set	Total	Failed	Non‐failed
1st cohort	602	298	304
Training	336	168	168
Validation	139	70	69
Test	127	60	67
2nd cohort external validation	1937	329	1608

All radiographs were offered by the Clinical and Radiographic Arthroplasty Register of Livio Sciutto Foundation Biomedical Research in Orthopaedic–ONLUS. The study was approved by the Institutional Ethical Committee (prot. 408/19, approved on 25 June 2019), Italy, and all patients gave their written informed consent.

### Image preprocessing

**Figure 1:**
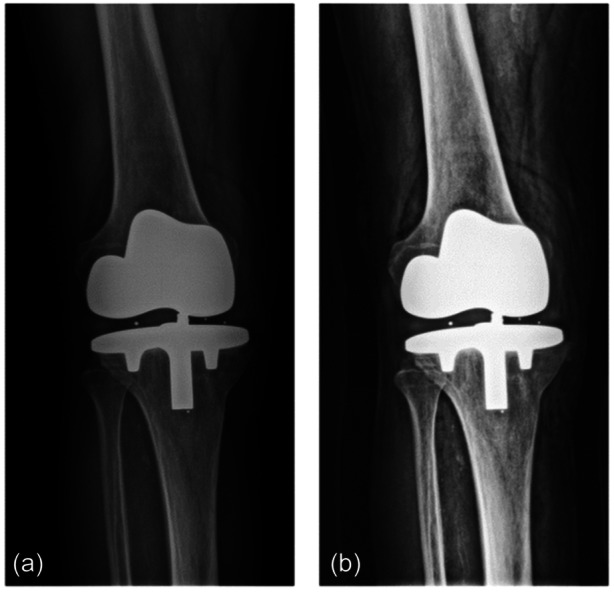
Image preprocessing. (a) Initial image. (b) Preprocessed image.

To ensure consistency among the images and provide good image resolution, images (DICOM format) underwent several preprocessing steps, as previously adopted for the development of the DL model of hip prosthesis failure [23]. Specifically, (i) a gamma power transformation was applied to reduce the mist‐like effect and increase brightness [24]; (ii) a sigmoidal function and the contrast‐limited adaptive histogram equalization (CLAHE) method were applied to enhance contrast, thus highlighting the prosthesis compared to bone structures [33]; (iii) a low pass‐filtering operation was performed using a two‐dimensional (2D) Gaussian smoothing kernel, eliminating frequencies above the cut‐off frequency, which typically represent noise. Finally, the image was resized to a standard input dimension (224 × 224) and normalized using *z*‐score standardization. Figure 1 shows an example of the initial and preprocessed images.

### Model development and testing

To develop the DL model for predicting TKA failure from conventional radiographs, transfer learning with fine‐tuning algorithms was applied, utilizing the pre‐existing hip prosthesis failure DL model [23]. Briefly, the hip prosthesis DL model consisted of a Densenet169 [9] pre‐trained for Imagenet [26], in which the Fully Connected layers of the original structure were replaced with a Global Average Pooling, a 128‐Dense, a Dropout and a 2‐Dense layers, and transfer learning fine‐tuning was applied [23]. To develop the TKA failure model, the first cohort of patients was considered, as detailed below, and the DL model was trained by freezing the layers of the pre‐trained hip prosthesis model until reaching the initial convolutional layer within the first dense block of the fourth stage. This transfer learning approach allows the model to be effectively trained on a relatively small dataset by leveraging the knowledge from pre‐trained models. Specifically, fine‐tuning was initiated from the layer named ‘conv4_block1_1_conv’ in the DenseNet architecture. This means that all layers up to and including this layer were frozen to preserve the pre‐trained weights, thereby maintaining the general features learned during the original training. Layers after ‘conv4_block1_1_conv’ were unfrozen and retrained to adapt to the TKA failure prediction task. This involved adding a Global Average Pooling layer, followed by a dense layer with 128 units and ReLU activation. A Dropout layer with a rate of 0.5 was included to prevent overfitting, and a final output layer with softmax activation was added to correspond to the number of output classes (two classes, in this case). The model was compiled using the Adam optimizer with a learning rate of 0.00009 and trained using categorical cross‐entropy loss. To enhance the model's generalization capabilities, data augmentation techniques such as rotation, shear, zoom and horizontal flipping were applied to the training images. The training data was batched with a size of 32 and shuffled to ensure a robust learning process. The model was trained over 150 epochs, with early stopping (patience of 25 epochs) and learning rate reduction on plateau (patience of 10 epochs and a reduction factor of 0.1) used as callbacks to prevent overfitting and optimize model performance.

The first cohort of patients was used for model development and internal testing. Data were split into training, validation and internal test sets using a patient‐stratified approach: 20% of the samples were reserved for model testing (127), and the remaining data were further divided into a 70%–30% split for training (336) and validation (139), respectively. The number of samples in the training, validation and test sets are reported in Table 1 for both failed and non‐failed groups. The second cohort, made of a different set of patients, served as external validation. The training and validation accuracy and loss along epochs were evaluated to assess the network performance. The model performance on the internal test and external validation sets was assessed by evaluating the sensitivity, specificity, accuracy and area under the curve (AUC). Specifically, considering the true positive (TP), false positive (FP), true negative (TN) and false negative (FN), the sensitivity is computed as TP/(TP + FN), the specificity as TN/(TN + FP) and the accuracy as (TP + TN)/(TP + TN + FP + FN).

## RESULTS

### Evaluation of model performance

**Figure 2:**
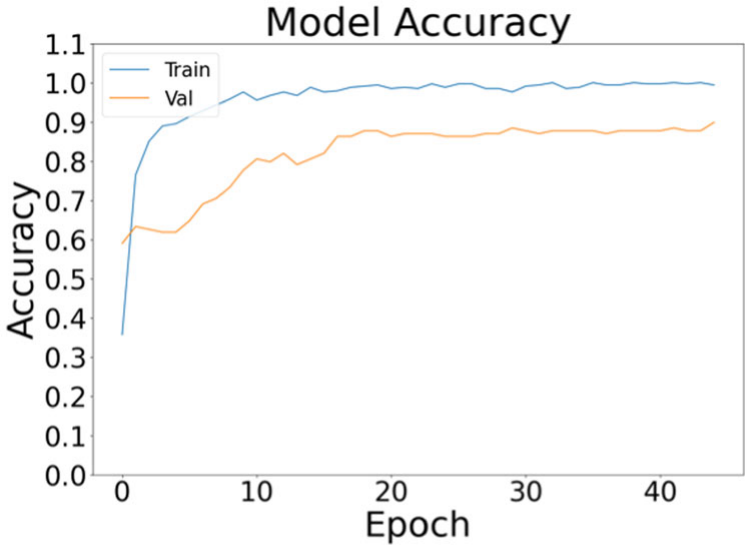
Trend of training (Train) and validation (Val) accuracy.

Figure 2 shows the training and validation accuracy as function of epochs (namely the iterations during training). The model's training accuracy, indicating how well the model fits the training data, reached a plateau around epoch 10 and a final value of 0.994. Similarly, the validation accuracy, which reflects the model's performance on unseen data during training, stabilized at around 15 epochs, achieving a value of 0.899. The loss metrics, which measure the error in predictions, also reached a plateau, with training loss stabilizing at epoch 27 and validation loss at epoch 17. The final loss values were 0.025 for the training set and 0.599 for the validation set. Lower loss values indicate better model performance, so the higher validation loss suggests that while the model performed extremely well on the training data, its performance on unseen data (validation) was slightly lower.

When applied to the test set, the model achieved a balanced accuracy of 0.85, demonstrating good performance in distinguishing between failed and non‐failed cases. Moreover, the model presented a sensitivity of 0.80 (i.e., it correctly identified 80% of the actual failed cases), specificity of 0.89 (i.e., it correctly identified 89% of the non‐failed cases) and AUC of 0.86, indicating an overall good ability to differentiate between the two classes across all thresholds. To further test the model's generalizability, it was applied to an external validation set. The performance metrics were slightly lower but still strong. Specifically, the model achieved a balanced accuracy of 0.79, sensitivity of 0.80, specificity of 0.78 and AUC of 0.86. Table 2 details the model performance in the validation, test and external validation sets.

**Table 2:** Performance in validation, test and external validation sets.

Metrics	Validation	Test	External validation
Balanced accuracy	0.899	0.848	0.790
Sensitivity	0.930	0.800	0.800
Specificity	0.870	0.895	0.780
AUC	0.938	0.860	0.860

Abbreviation: AUC, area under the curve.

**Figure 3:**
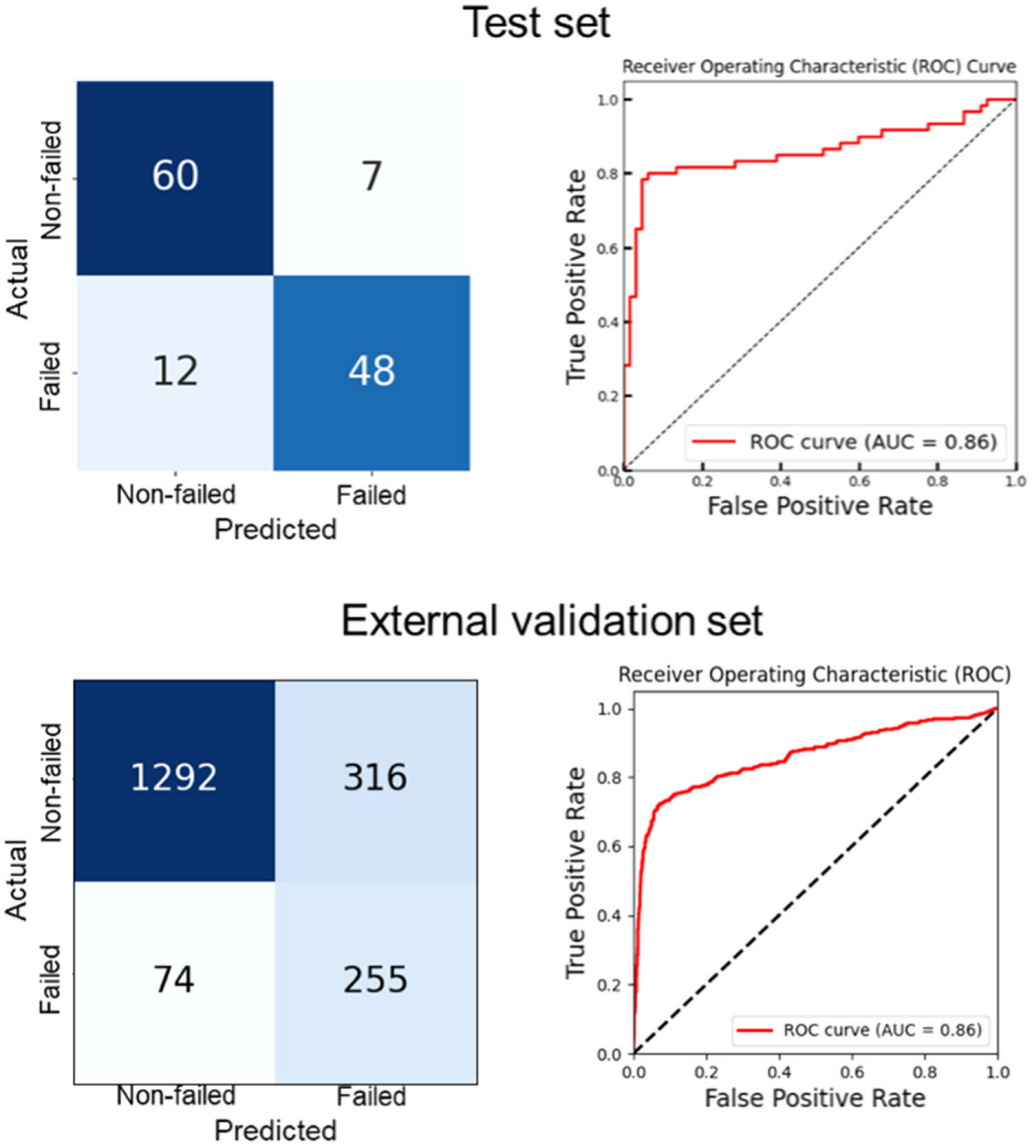
Model performance in the test set (top) and external validation set (bottom).

Figure 3 shows the confusion matrix and the receiver operating characteristic curve (ROC) for the test (top) and external validation (bottom) sets. Within the test set, 48 images were correctly classified as failed, with a probability of 0.999 ± 0.003 and 60 images were correctly classified as non‐failed, with a probability of 0.965 ± 0.066. Within the external validation set, 255 images were correctly classified as failed and 1292 images were correctly classified as non‐failed.

**Figure 4:**
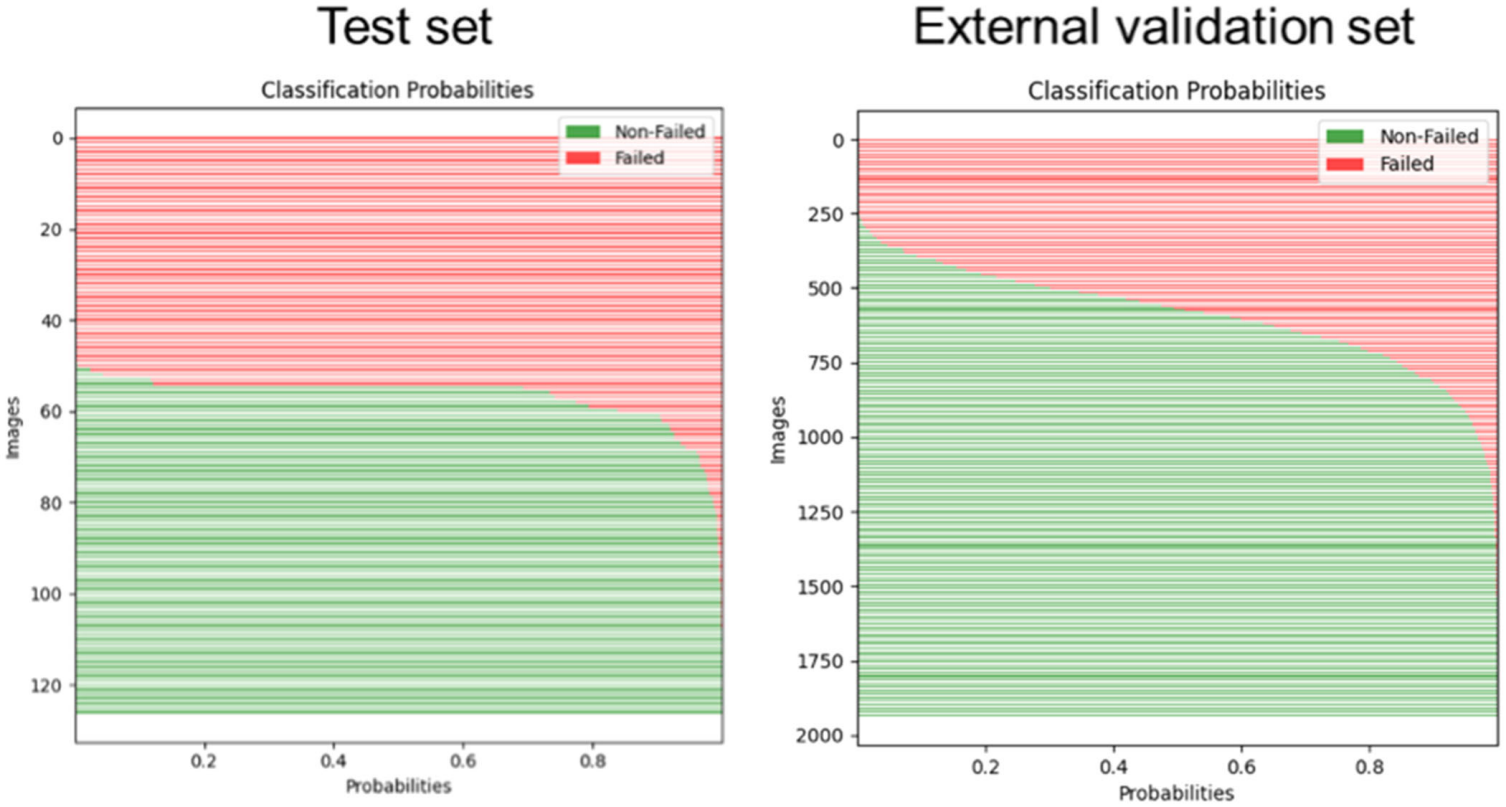
Classification probabilities in the test set (left) and external validation set (right).

Figure 4 shows the predicted probabilities for the test and external validation sets

## DISCUSSION

The DL model developed to predict TKA failure from post‐operative radiographs demonstrated an excellent performance in the internal test, and showed a moderately accurate AUC, which remains clinically relevant [5], in the external validation phase. The model's high sensitivity, exceeding its specificity, aligns with the goal of the study of developing an accurate screening method for early detection of TKA failure during post‐operative follow‐up. As follow‐up progresses, however, also the specificity becomes increasingly important. The findings of the present study also highlight the feasibility and advantages of using transfer learning. By leveraging a pre‐trained DenseNet model and fine‐tuning it for TKA failure prediction, a previously developed image‐based DL model was successfully adapted to a new but related task. This approach not only demonstrated strong performance but also underscored the efficiency of transfer learning in maximizing the utility of existing models, even with limited data.

Currently, the standard approach for evaluating knee replacements relies on traditional radiographic imaging, primarily aimed at detecting issues such as component misalignment, subsidence, prosthesis loosening, and polyethylene wear. However, early detection of these complications using 2D radiographs poses significant challenges for clinicians, prompting efforts to develop computer‐based image analysis techniques.

Several ML models have been proposed, predicting various outcomes of TKA [6, 8, 11, 12, 13] and, to the best of the authors' knowledge, only two DL models were proposed to predict TKA failure from conventional radiographs. Although these models produced promising results, they focused solely on TKA loosening. Shah et al. [27] developed an ML model to detect THA and TKA loosening by combining radiographic and clinical data. The joint data model reached an overall accuracy of 88.3% on the test set, whereas, considering TKA patients only, the balanced accuracy was 85.8% and the sensitivity of 69.8%. Compared to Shah et al.'s results, the current algorithm demonstrates a higher sensitivity, indicating a greater ability to detect patients with failed TKA. Additionally, the DL model in this study was applied to images of patients with both successful and failed implants, whereas Shah et al. exclusively included patients undergoing primary hip or knee revision arthroplasty, using intraoperative findings of fixed or loose implants as the gold standard for diagnosis. In another study, Lau et al. [16], developed an ML model capable of detecting loosening with high accuracy, achieving an AUC (93.5%) in the internal validation set. However, despite its strong performance, this model was limited to detecting implant loosening, unlike the current study, which considers TKA failure as the need for revision surgery. Moreover, Lau et al. compared the performance of their ML model on radiographic data alone versus a combination of radiographic and patient's characteristics, finding that the model outperformed with radiographic data alone.

Concerning the limitations of this study, the first one is the small sample used for the first cohort. Furthermore, a trait that has been thought of as a strength during the design of the study is that the concept of ‘TKA failure’ coincides with the necessity to undergo revision surgery. Nonetheless, this could also be a limitation. Indeed, even though most cases of failure were due to implant loosening, which is clearly visible on radiographs, and hence distinctly detectable by the DL model, the choice to perform a TKA revision can depend on different factors, regardless of the knee radiographic presentation. For example, implant failure due to infection or pain might present as a fixed and well‐positioned prosthesis on radiographs, potentially leading the DL model to misclassify images of failed TKA that do not appear loosened. Thus, the model may struggle to differentiate between radiographically similar images of failed and non‐failed TKA. Other misclassifications can be attributed to the DL model intrinsic activity. For instance, Grad‐CAM heatmap analysis, which allows for visual representation of the model functioning, revealed that in some cases, the model incorrectly classified failed prosthesis as non‐failed because it focused on image areas without pathological features.

Despite these limitations, this study demonstrated several strengths and innovations. Herein, it was developed a novel image‐based DL model to predict TKA failure from post‐primary TKA radiographs, where ‘failure’ is defined as the need for revision surgery, extending beyond mere loosening. This approach is clinically relevant because TKA failure is not solely associated with loosening, and the model aims to reflect this broader reality. Moreover, it was successfully demonstrated, for the first time, the effectiveness of a transfer learning fine‐tuning approach from a previously developed DL model for hip prosthesis failure. This approach enabled the achievement of satisfactory results, consistent with existing literature, with a relatively small dataset. Importantly, the model's performance was validated not only internally but also through external validation, which tested the model's reliability and generalizability across different datasets and settings.

In the context of hip failure prosthesis prediction, clinical variables have proven to be effective predictors [3]. Thus, future work should focus on integrating patients' clinical information with the image‐based DL model to enhance diagnostic performance. Moreover, to further confirm the generalizability of the developed model, the DL pipeline should be tested on a larger multi‐centric cohort of patients. Finally, it is important to note that, due to the retrospective nature of our study, the study was limited to radiographs only. Radiographs are the most common imaging modality in knee arthroplasty, particularly in routine pre‐operative and post‐operative evaluations, due to their wide availability, lower cost, and reduced radiation exposure. While radiographs are effective for assessing general alignment and implant positioning, they do not provide the same level of detail as computed tomography (CT) scans. CT scans offer more precise three‐dimensional imaging and better visualization of the bone‐implant interface, which can be crucial in certain complex cases or when planning for revision surgeries. Although CT is not as commonly used as radiographs due to higher costs and radiation exposure, the developed pipeline can be applied to other imaging modalities, including CT scans, to enhance diagnostic capabilities when multiple imaging modalities are considered.

The application of this DL model can find its place in the scenario of ‘virtual clinics’, where the combination of artificial intelligence algorithms and online clinical appointments can result in the realization of a tailored schedule of follow‐up. Pilot studies by Wood et al. [31] and Marsh et al. [21, 22] have proposed virtual clinics for follow‐up based on the combination of patient‐reported outcome measures and radiographs, which resulted in fewer appointments and reduced travels for patients [20]. In this way, the prompt identification of asymptomatic patients in need of revision surgery can help prevent late‐stage, complex revision surgeries, reducing risks of morbidity and poor functional outcomes for the patient, as well as high management costs for the National Health System.

## CONCLUSIONS

The present study demonstrated the effectiveness of the developed DL model in detecting TKA failure from post‐primary TKA radiographs, with moderate accuracy in predicting the likelihood of future revision surgery. The model's strong performance in both internal and external validation phases indicates its potential utility in clinical settings, paving the way for innovative solutions in orthopaedic care. Additionally, the study highlighted the potential to transfer knowledge from a DL model originally designed for detecting THA failure to the identification of knee prosthesis failure, thereby enhancing the model's generalization capabilities.

## AUTHOR CONTRIBUTIONS

All authors have made substantial contributions to all of the following: (1) the conception and design of the study, or acquisition of data, or analysis and interpretation of data, (2) drafting the article or revising it critically for important intellectual content and (3) final approval of the version to be submitted.

## CONFLICT OF INTEREST STATEMENT

Mattia Loppini declares Research grant as principal investigator (2022YME9N3) from the Italian Ministry of University and Research; Research grant as co‐principal investigator (GR‐2019‐12371158) from the Italian Ministry of Health; Research grants as principal investigator for postmarket study formedical devices from Zimmer Biomet; Financial support for attending symposia and educational programs from Zimmer Biomet; Scientific Director of Fondazione Livio‐Sciutto. Valentina Corino declares Research grant as a co‐prinvipal investigator (2022YME9N3) from the Italian Ministry of University and Research. The remaining authors declare no conflicts of interest.

## ETHICS STATEMENT

The study was conducted in accordance with the Declaration of Helsinki, and approved by the Ethics Committee of Humanitas Research Hospital (protocol code: ICH‐ORT‐2019‐01 on 16 May 2019). The authors allow publication of this manuscript.

## Data Availability

The data supporting the reported results can be found in a repository (Zenodo).
